# The impact of physical activity and exercise interventions for physical health in people with cystic fibrosis: protocol for a systematic review

**DOI:** 10.1186/s13643-021-01614-8

**Published:** 2021-02-26

**Authors:** Owen W. Tomlinson, Sarah Denford, Alan R. Barker, Jane E. Schneiderman, Emma S. Campisi, Helen Douglas, Sarah Rand, Melitta A. McNarry, Kelly A. Mackintosh, Craig A. Williams

**Affiliations:** 1 Children’s Health and Exercise Research Centre, Sport and Health Sciences, University of Exeter, Heavitree Road, Exeter, EX1 2LU UK; 2 Royal Devon and Exeter NHS Foundation Trust Hospital, Barrack Road, Exeter, EX2 5DW UK; 3 Centre for Public Health and Epidemiology, Population Health Sciences, Bristol Medical School, University of Bristol, Bristol, BS8 2PS UK; 4 Division of Respiratory Medicine, The Hospital for Sick Children, 555 University Avenue, Toronto, Ontario M5G 1X8 Canada; 5 University of Toronto, 27 King’s College Circle, Toronto, Ontario M5S 1A1 Canada; 6 UCL Great Ormond Street Institute of Child Health, 30 Guilford Street, London, WC1N 1EH UK; 7 Great Ormond Street Hospital for Children NHS Foundation Trust, Great Ormond Street, London, WC1N 3JH UK; 8 Applied Sports, Technology, Exercise and Medicine Research Centre, College of Engineering, Bay Campus, Swansea University, Fabian Way, Swansea, SA1 8EN UK

**Keywords:** Pulmonary disease, Movement, Lifestyle, Healthcare

## Abstract

**Background:**

Cystic fibrosis (CF) is a genetically inherited, life-limiting condition, affecting ~90,000 people globally. Physical activity (PA) and exercise form an integral component of CF management, and have been highlighted by the CF community as an area of interest for future research. Previous reviews have solely focused on PA or structured exercise regimens independent of one another, and thus a comprehensive assessment of the physical health benefits of all PA, including exercise, interventions, is subsequently warranted. Therefore, the purpose of this review is to evaluate the effects of both PA and exercise upon outcomes of physical health and healthcare utilisation in people with CF.

**Methods:**

A systematic review has been registered and reported in line with Preferred Reporting Items for Systematic Reviews and Meta-Analysis-P guidelines. This will include randomised control trials on the effects of PA and exercise, relative to usual treatment, upon people with CF. Primary outcomes will include variables associated with fitness, PA, lung health, inflammation, body composition, glycaemic control and patient-reported outcomes. Secondary outcomes will include adverse events and healthcare utilisation. Searches will be undertaken in Ovid MEDLINE, OVID EMBASE, PsychINFO, ERIC, SPORTDiscus, ASSIA, CCTR, CINHAL and Web of Science databases, and will be searched from date of inception onwards. Two reviewers will independently screen citations and abstracts, and full-texts, for inclusion and data extraction, respectively. Methodological quality will be assessed using the Cochrane Risk of Bias-2 tool. If feasible, random-effects meta-analyses will be conducted where appropriate. Additional analyses will explore potential sources of heterogeneity, such as age, sex, and disease severity.

**Discussion:**

This systematic review will build on previous research, by comprehensively assessing the impact of both PA and exercise upon physical health and healthcare utilisation in people with CF. Results of this review will be utilised to inform discussions that will ultimately result in a consensus document on the impact of physical activity and exercise for people with CF.

**Systematic review registration:**

PROSPERO CRD42020184411

**Supplementary Information:**

The online version contains supplementary material available at 10.1186/s13643-021-01614-8.

## Introduction

Cystic fibrosis (CF) is a genetically inherited condition which affects multiple organ systems. Disease progression is predominantly observed through deteriorating lung function [1]. Currently, there are ~90,000 people globally with CF [2], the majority of whom are in Europe [3] and North America [4, 5]. Substantial growth in the size of the CF population is anticipated [6], accompanied by an increase in life expectancy into the fifth decade of life [7]. Presently, there is no cure for CF, and therefore it is a life-long condition that is ‘managed’ as opposed to ‘cured’. Whilst a number of promising pharmacological advances have been made [8], CF is still fundamentally managed using a combination of medication, nutrition, physiotherapy and physical activity (PA), or more specifically, exercise [9].

The outcomes of a recent patient-driven research priority partnership [10], highlighted the need for research to advance our understanding of the benefits of PA and exercise [11], and simplify treatment burden in CF [10]. Previously, the time spent being physically active [12, 13], as well as the associations between PA and health [13] and the effect of PA [14] and structured exercise interventions [15] for CF, has been systematically reviewed. These reviews have concluded that individuals with CF spent a similar amount of time being physically active relative to non-CF peers [12, 13], and that despite heterogeneity in study designs, interventions and outcomes, there was no evidence to actively discourage PA or exercise in CF [15].

However, despite being reviewed independently previously, PA and exercise are not mutually exclusive constructs. Exercise is a structured subcomponent of PA conducted for the inherent health associations. Nonetheless, evidence suggests that all PA, irrespective of purpose or intensity, is associated with improved health status in CF [12–15]. Therefore, both PA and exercise must be considered when attempting to integrate activity into the daily lives of those with CF, and not solely the prescription of structured exercise per se. Consequently, an updated review that simultaneously, and universally, accounts for all aspects of PA, including exercise, is warranted.

The main objective of this systematic review is to identify the effect of both PA and exercise upon parameters of physical health and healthcare utilisation, relative to usual care, in people with CF. In addition, a secondary objective is to identify if different effects are present in people of differing age, sex, and disease status, and whether certain components of interventions are linked to favourable outcomes in people with CF (e.g. delivery method, modality, intensity, frequency, length).

## Methods

This review has been designed by experts in PA, exercise science and the physiotherapy management of CF. The present protocol has been registered on the PROSPERO database (CRD42020184411) and is being reported in accordance with the reporting guidance provided in the Preferred Reporting Items for Systematic Reviews and Meta-Analysis–Protocol (PRISMA-P) statement [16, 17] (see checklist in Additional file 1). If any updates to the protocol are required during the process of undertaking the review, these will be appropriately updated on the PROSPERO database, and detailed in the subsequent systematic review to be published.

### Eligibility criteria

Studies will be limited to those published in English. No restrictions will be placed on publication dates. Studies will be included in this systematic review based upon a series of pre-planned inclusion and exclusion criteria for the following domains:

#### Participants

This review will solely include individuals with a clinical diagnosis of CF [18]. If studies include people with CF as part of a wider population (e.g. people with a pulmonary disease), results will be included in the systematic review provided information for the CF participants can be successfully retrieved in isolation from other non-CF groups. If CF-specific data cannot successfully be retrieved from published manuscripts, study authors will be contacted for data. There is no restriction on age.

#### Interventions

Studies must include any intervention based on promoting PA, sport, exercise, recreation, or movement. Given that multiple factors can be considered when describing interventions [19], and the generally complex and broad nature of PA and exercise interventions, there exists a possibility of inadvertently excluding studies if explicit interventions are defined in advance. Therefore, no criteria related to time frame, location, setting or delivery provider will be used to limit inclusion and thus maximise potential inclusion of eligible studies. Interventions where PA and/or exercise form a secondary sub-component of a wider intervention (e.g. nutritional, educational, pharmacological) will be excluded from this review if the effects of PA/exercise alone cannot be successfully isolated and retrieved. If exercise-specific data cannot successfully be retrieved from published manuscripts, study authors will be contacted for data.

#### Comparison

Primarily, interventions will be compared against patients receiving their usual clinical care (i.e. no intervention). Secondly, studies that compare two intervention arms within a single cohort of people with CF (e.g. high intensity vs. low intensity exercise) will also be included in an effort to identify dose-response effects.

#### Outcomes

Factors associated with physical health and healthcare utilisation will be included in the review, but will not be explicitly searched for upon the basis of the following outcomes. As stated, previous reviews [13, 15] have identified heterogeneity in the variables that have been reported and therefore outcomes will be obtained at the extraction stage, provided that the intervention has met the stated criteria above. It is anticipated that the primary outcomes will be related to (1) fitness: including, but not limited to, muscle strength, aerobic fitness and walking distance; and (2) physical activity (objective and subjective outcomes): including, but not limited to, total energy expenditure, step count and time spent in light, moderate and vigorous physical activity; (3) lung health: including, but not limited to, forced expiratory volume in 1 s (FEV_1_), forced vital capacity (FVC), tiffeneau index (FEV_1_/FVC), peak expiratory flow (PEF) and lung clearance index (LCI); (4) inflammation: including, but not limited to, C-reactive protein (CRP) and cytokines such as interleukin 6 (IL-6) and 8 (IL-8); (5) body composition: including, but not limited to, fat mass, fat-free mass, body mass index (BMI) and bone mineral density; (6) glycaemic control: including, but not limited to, blood glucose levels such as glycated haemoglobin (HbA1c); (7) patient-reported outcome measures: including, but not limited to, quality of life and its components, breathlessness and fatigue. It is anticipated secondary outcomes will be related to (1) serious adverse events: which may take multiple forms such as sprains, strains, fractures, haemoptysis, exacerbations and desaturation; (2) healthcare utilisation: which may take multiple forms, such as inpatient hospital days, medication usage and healthcare costs.

#### Study design

This systematic review will be limited to randomised control trials (RCT) comparing PA and/or exercise interventions (as above) to standard CF care (i.e. no intervention), and/or another PA/exercise intervention.

### Information sources and search

The following electronic databases will be searched: Ovid MEDLINE, OVID EMBASE, PsychINFO, ERIC, SPORTDiscus, ASSIA, CCTR, CINHAL, and Web of Science. These databases will be searched from respective dates of inception onwards. Grey literature will not be included to ensure quality standards are met. The literature searches will be initially designed by the research team, and conducted by an information specialist, who will also customise the search for each database. The search will include a broad range of terms and keywords related to PA, exercise and RCTs. The search terms have been restricted to ‘Population/Intervention/Comparison/Outcome’ (PICO) domains of participants, intervention, and study design. Given the wide heterogeneity in outcome variables available, and the way they are reported as has been previously explained, these have been omitted from the search strategy in order to increase returns. A draft search strategy, utilising these domains, is provided in Table 1.

**Table 1:** Draft search strategy

Domain	Terms
Population	cystic fibrosis OR CF
Intervention	physical activ* OR exercis* OR sport* OR recreation* OR move* OR yoga OR Tai Chi OR walk* OR run OR runn* OR play* OR jog* OR cycl* OR game* OR inactive* OR sedentary OR swim* OR hike OR hiking* OR fitness OR gym* OR resistance OR aerobic OR leisure time OR active travel OR jumping OR danc*
Study design	random* OR control trial OR RCT OR clinical trial OR randomly OR groups OR allocat* OR crossover OR (((systematic OR state-of-the-art OR scoping OR literature OR umbrella) ADJ (review* OR overview* OR assessment*)) OR “review* of reviews” OR meta-analy* OR metaanaly* OR ((systematic OR evidence) ADJ1 assess*) OR “research evidence” OR metasynthe* OR meta-synthe*).tw. OR exp Review Literature as Topic/ OR exp Review/ OR Meta-Analysis as Topic/ OR Meta-Analysis/ OR “systematic review”/

Records will be imported and managed via online evidence synthesis software (Covidence systematic review software, Veritas Health Innovation, Melbourne, Australia).

### Data selection and collection process

All articles returned from searches will be screened by two independent researchers. First, titles and abstracts of identified papers will be assessed in relation to aforementioned eligibility criteria. Second, eligible articles will have full-texts retrieved and then screened in full, again against the aforementioned eligibility criteria. If necessary, any disagreements that arise will be resolved via discussion with a third reviewer. A flow chart, detailing inclusion, and exclusion of studies at each stage, will be included in the final, published review.

#### Data extraction

Data will be extracted independently by two reviewers using a standardised data extraction template designed for this purpose (Additional file 2). Data will be extracted on the following: intervention design and delivery (including, but not limited to location, modality, intensity, frequency, length of intervention), participant characteristics (sex, age and disease severity of both control and intervention group(s)), and outcomes (variables, and magnitude of change from baseline for continuous data). Disagreements will be resolved via discussion with a third reviewer if necessary. This data extraction will be piloted on five randomly selected papers by two independent authors. If extracted results are in agreement, this extraction template will be uniformly utilised by all authors.

The majority of outcomes produce objective measures, and these will be prioritised over subjective measures where possible. Data will be extracted based upon both (where possible): (1) absolute differences in outcomes at follow up; and (2) differences between groups (i.e. intervention vs. control) at follow-up. This will allow for assessment of data if studies report outcomes in differing formats.

#### Risk of bias assessment

Risk of bias (RoB) of individual studies will be assessed using the RoB2 Tool for RCTs [20]. Assessments will be made by two reviewers independently, with any disagreements being resolved via discussion with a third reviewer when necessary. Studies identified as being at high risk of bias will be included, although the quality of each study will be presented in results and will also be narratively discussed.

### Synthesis

Data synthesis will occur in several stages. Initially, summary tables will be created to detail characteristics of each study included in the final review. This will include the aforementioned data to be extracted using Additional file 2 (intervention design and delivery, participant characteristics and outcomes). Absolute differences in outcomes at follow-up, and mean differences between groups (i.e. intervention vs. control) will be reported in tables, as well as standardised mean differences for outcomes that are reported in more than one way (e.g. FEV_1_ as L, or %_predicted_). Additional narrative discussion will also be provided.

Secondly, meta-analyses will be undertaken for primary outcomes where possible, using data pooled from each study. Since heterogeneity is expected a priori, we will estimate the pooled effect and its 95% confidence interval using the random effects model, which assumes the study effects follow a normal distribution, considering both within- and between-study variation. Pooled effect sizes, using Hedges *g*, will be interpreted with reference to Cohen’s thresholds [21]: trivial (<0.2), small (0.2 to <0.5), moderate (0.5 to <0.8) and large (≥0.8); whereby positive effect size values indicate higher scores of the outcome in favour of the PA/exercise group. All secondary outcomes, and non-continuous primary outcomes (e.g. categorical data), will be reported using Synthesis Without Meta-analysis (SWiM) guidelines [22].

If studies have two CF groups (e.g. different exercise intensities or protocols), multilevel models will be used as their data will be analysed independently with the control group, thus yielding multiple effect sizes for those studies and outcomes. Both research study and intra-study groups will be included as random effects in the model. Cluster robust estimates will be produced, weighted by inverse sampling variance to account for the within- and between-study variance (tau-squared). Restricted maximal likelihood estimation will be used in all models.

Finally, meta-regression will be utilised to determine differences between sub-groups based upon disease severity (FEV_1%_ categories: ≥70, 40-69, <40), age (<18 years, ≥18 years) and sex (male, female), all of which are significant predictors for long-term outcomes and survival in CF [7, 23]. Moreover, the impact of differing delivery methods, modalities, intensities, frequencies, and lengths of interventions will also be investigated via meta-regression. Analyses will be contingent on a sufficient number of studies (≥10) being found [24]. These analyses will use age, sex and disease severity as moderators of PA or exercise. If appropriate (i.e. a sufficient ratio of studies to co-variates are found), multilevel models will be produced for each sub-group (e.g. sex, age, FEV_1_ category) and a fixed-effects with moderators model used to compare the models to ascertain whether there was a significant difference (*p*<0.05). Sensitivity analyses will be performed using the leave-one-out method to examine the impact of removal of individual effect sizes. Heterogeneity will again be examined through the *I*^2^ statistic, with *I*^2^>50% indicating ‘substantial’ heterogeneity [24]. If meta-regression is not possible due to insufficient power, the sub-group analyses will be undertaken, based upon aforementioned categories of disease severity, age and sex. If quantitative syntheses are not possible for determining differences between sub-groups and intervention methods, the aforementioned SWiM guidelines [22] will be utilised to report findings.

All meta-analyses will be undertaken using RevMan (Review Manager v5.4; The Nordic Cochrane Centre, The Cochrane Collaboration, Copenhagen, Denmark) and Stata (Stata v16; StataCorp LLC, College Station TX, USA) software programmes.

### Meta-bias

To determine whether publication bias is present, it will be examined using Egger’s linear regression test for funnel plot asymmetry [25], and graphically presented by contour-enhanced funnel plots with Duval and Tweedie’s trim and fill used.

### Grading of evidence

Certainty of evidence for outcomes will be judged using Grading of Recommendations Assessment, Development and Evaluation (GRADE) methodology [26, 27]. This will be undertaken by two independent reviewers, who will examine the study limitations, publication bias, imprecision, inconsistency and indirectness. The evidence will then be classified as high, moderate, low or very low.

## Discussion

It is well established that PA and exercise, are integral components in the management of CF [9], and consultation with the CF community has identified exercise as a research priority [10]. However, the specific effects of PA and/or exercise interventions on physical markers of health in CF have not been fully quantified to date. Indeed, previous reviews have solely focused on structured exercise [15]. Whilst such reviews are useful, it is important to ensure that all interventions that target improvements in habitual PA, exercise programmes that do not have set structures, or generalised increases in movement away from a prescriptive framework, are incorporated. Therefore, the results of this systematic review will pool findings of high-quality studies, by only extracting RCTs, to determine true effects of PA and exercise interventions in this patient population. Whilst RCTs tend to result in higher quality evidence, by only including RCTs within the protocol and therefore omitting observational and non-randomised control studies, it is feasible that the effect of PA, or exercise, on some markers of physical health and healthcare utilisation may not be established. Moreover, the broad range of ways in which PA and exercise interventions can be implemented, such as differing modalities, locations, frequency, intensity, materials and procedures [19] may result in an under-powering of meta-analyses due to an inability to pool data from independent studies. Whilst this may initially be perceived as a limitation, this could simultaneously provide the impetus for researchers and clinicians to standardise future interventions to determine true effects of each component of delivery.

Whilst this systematic review is focused on outcomes related to physical health and healthcare utilisation, the importance of PA, or exercise, for mental health in CF should not be ignored or understated. Therefore, a separate systematic review has been developed to establish the effects of PA and exercise upon parameters of mental health in CF (PROSPERO: CRD42019151034). The results of these systematic reviews, focusing on physical and mental health outcomes, will then be utilised to inform discussions amongst an international panel of experts in exercise and CF, to create a consensus document on the impact of PA and exercise for people with CF. Both the findings of the present systematic review, and the anticipated consensus document, will be disseminated via conference presentations and peer-reviewed academic journals.

In summary, by establishing the effect, and the associated magnitude, of any PA, or exercise intervention, findings can influence guidelines and consensus documents that are utilised by clinical teams in daily practice. Thus, ultimately, such a systematic process can, in turn, enhance the care of people with CF.

## Supplementary Information

**Additional file 1.** PRISMA-P Checklist.

**Additional file 2.** Data Extraction Form.

## Data Availability

Data and materials will be made available from the Open Science Framework (doi: 10.17605/OSF.IO/SFGJQ).

## Abbreviations

BMI: Body mass index
CF: Cystic fibrosis
CRP: C-reactive protein
FEV_1_: Forced expiratory volume in 1 s
FVC: Forced vital capacity
GRADE: Grading of Recommendations Assessment, Development and Evaluation
HbA1c: Glycated haemoglobin
IL-6: Interleukin 6
IL-8: Interleukin 8
LCI: Lung clearance index
PEF: Peak expiratory flow
PICO: Population/Intervention/Comparison/Outcome
PRISMA: Preferred Reporting Items for Systematic Reviews and Meta-Analysis
PA: Physical activity
RoB: Risk of Bias
RCT: Randomised control trial
SWiM: Synthesis Without Meta-analysis
95% CI: 95% confidence interval
