# Supplementary material for: The impact of physical activity and exercise interventions for physical health in people with cystic fibrosis: protocol for a systematic review
Source: Syst Rev. 2021 Feb 26;10:64. doi: 10.1186/s13643-021-01614-8 (PMC7913325; doi:10.1186/s13643-021-01614-8)
Supplement: Supplementary file 2 — Additional file 2. Data Extraction Form. [file 13643_2021_1614_MOESM2_ESM.pdf]

Appendix 2: Data Extraction Forms.

‘Exercise and physical activity for physical health in cystic fibrosis’

PROSPERO CRD42020184411

**Date form completed** *(dd/mm/yyyy)*		**Name of person extracting data**	
**Date form checked** *(dd/mm/yyyy)*		**Name of person checking data**	

1. Study Details

**Covidence ID**	
**Study ID** *(surname of first author and year first full report of study was published e.g. Smith 2001)*	
**Report ID** *(if different to Study ID)*	**Report IDs of other reports of this study** *(e.g. duplicate publications, follow-up studies)*
Title	
Reference citation	
Study author contact details	
Publication type *(e.g. full report, abstract, letter)*	

2. Study Eligibility

Study Characteristic	Eligibility criteria	Eligibility criteria met?			Page no.
		Yes	No	Unclear	
Participants	Does this include patients with cystic fibrosis?	☐	☐	☐	
Intervention	Does this include an exercise and/or physical activity intervention?	☐	☐	☐	
Study Design	Is this study a randomised control trial?	☐	☐	☐	
**INCLUDE** ☐ **QUERY** ☐ **EXCLUDE** ☐					
Reason for exclusion (tick more than one if necessary)	Does not include people with cystic fibrosis	☐			
	Does not include exercise/PA intervention	☐			
	Is not a randomised control trial	☐			

DO NOT PROCEED IF STUDY IS EXCLUDED FROM REVIEW

3. Study Data

**Study Characteristic**	**Data**		**Location in text (page no./table no.)**
Methods	Brief aim of study:<br>*(e.g. To investigate running intervention on severe CF)*		
	Intervention Design<br>*(e.g. Gym based; running; 50% max heart rate; 3 times/week; 12 weeks)*	Location:<br>Modality:<br>Intensity:<br>Frequency:<br>Length:	
	Control Group<br>*(e.g. usual care CF)*		
	Single centre/multi-centre(n)?<br>*(e.g. Single UK CF centre; 12 centres in 3 countries)*		
	Country/countries?<br>*(e.g. UK, USA, Italy)*		
	Dates patients recruited:<br>*(e.g. 01/01/2002-31/12/2002)*		
	How were patients randomised?<br>*(e.g. simple, block, stratified, 1:1, 2:2)*		

<table>
<tr><td rowspan="13">Participants</td><td colspan="4">Inclusion criteria:

Exclusion criteria:</td><td></td></tr>
<tr><td></td><td>Total</td><td>Intervention</td><td>Control</td><td rowspan="6"></td></tr>
<tr><td>Recruited (n)</td><td></td><td></td><td></td></tr>
<tr><td>Baseline Tested (n)</td><td></td><td></td><td></td></tr>
<tr><td>Completed Intervention (n)</td><td></td><td></td><td></td></tr>
<tr><td>Completed follow-Up (if applicable) (n)</td><td></td><td></td><td></td></tr>
<tr><td>Analysed (n)</td><td></td><td></td><td></td></tr>
<tr><td></td><td>Total</td><td>Intervention</td><td>Control</td><td rowspan="5"></td></tr>
<tr><td>Age (mean ± SD)</td><td></td><td></td><td></td></tr>
<tr><td>Sex (male: n, %)</td><td></td><td></td><td></td></tr>
<tr><td>Genotype (ΔF508/ ΔF508: n, %)</td><td></td><td></td><td></td></tr>
<tr><td>FEV_1 Category*(mild/mod/severe, n)</td><td></td><td></td><td></td></tr>
<tr><td colspan="4"></td></tr>
<tr><td>Outcomes
(please label numerically to align with results below)</td><td colspan="2">Parameter (e.g. FEV_1, exercise tolerance)
1.
2.
3.</td><td colspan="2">Measurement Scale (e.g. litres, %pred, VO_{2max})
1.
2.
3.</td><td></td></tr>
<tr><td>Results
(As per numerical</td><td>Change from baseline (intervention)
1.</td><td colspan="2">Change from baseline (control)

1.</td><td>Intervention vs. Control difference post-intervention
1.</td><td></td></tr>
</table>

assignment above)	2.<br>3.	2.<br>3.	2.<br>3.	
Follow-up Length (if applicable)				
Conflicts of Interest				
Notes of interest				
Missing information				

*FEV_1 Categories are as follows: Mild ≥70%, Moderate 40-69%, Severe <40%.
